# Requirement of Stat3 Signaling in the Postnatal Development of Thymic Medullary Epithelial Cells

**DOI:** 10.1371/journal.pgen.1005776

**Published:** 2016-01-20

**Authors:** Rumi Satoh, Kiyokazu Kakugawa, Takuwa Yasuda, Hisahiro Yoshida, Maria Sibilia, Yoshimoto Katsura, Ben Levi, Jakub Abramson, Yoko Koseki, Haruhiko Koseki, Willem van Ewijk, Georg A. Hollander, Hiroshi Kawamoto

**Affiliations:** 1 Laboratory for Lymphocyte Development, RIKEN Research Center for Allergy and Immunology, Yokohama, Japan; 2 Laboratory for Developmental Genetics, RIKEN Center for Integrative Medical Sciences, Yokohama, Japan; 3 Laboratory for Immune Crosstalk, RIKEN Center for Integrative Medical Sciences, Yokohama, Japan; 4 Laboratory for Immunogenetics, RIKEN Center for Integrative Medical Sciences, Yokoham, Japan; 5 Department of Medicine I, Institute of Cancer Research, Comprehensive Cancer Center, Medical University of Vienna, Vienna, Austria; 6 Division of Cell Regeneration and Transplantation, Advanced Medical Research Center, Nihon University School of Medicine, Tokyo, Japan; 7 Department of Immunology, Weizmann Institute of Science, Rehovot, Israel; 8 Department of Molecular Cell Biology and Department of Immunology, Leiden University Medical Center, RA Leiden, the Netherlands; 9 Laboratory of Pediatric Immunology, Center for Biomedicine, University of Basel, and the University Children’s Hospital, Basel, Switzerland; 10 Laboratory of Developmental Immunology Weatherall Institute of Molecular Medicine and Department of Paediatrics, University of Oxford, Oxford, United Kingdom; 11 Department of Immunology, Institute for Frontier Medical Sciences, Kyoto University, Kyoto, Japan; University of Edinburgh, UNITED KINGDOM

## Abstract

Thymic medullary regions are formed in neonatal mice as islet-like structures, which increase in size over time and eventually fuse a few weeks after birth into a continuous structure. The development of medullary thymic epithelial cells (TEC) is dependent on NF-κB associated signaling though other signaling pathways may contribute. Here, we demonstrate that Stat3-mediated signals determine medullary TEC cellularity, architectural organization and hence the size of the medulla. Deleting Stat3 expression selectively in thymic epithelia precludes the postnatal enlargement of the medulla retaining a neonatal architecture of small separate medullary islets. In contrast, loss of Stat3 expression in cortical TEC neither affects the cellularity or organization of the epithelia. Activation of Stat3 is mainly positioned downstream of EGF-R as its ablation in TEC phenocopies the loss of Stat3 expression in these cells. These results indicate that Stat3 meditated signal via EGF-R is required for the postnatal development of thymic medullary regions.

## Author Summary

Thymic medulla is known to be an essential site for the deletion of auto-reactive T cells. Whereas it has been well documented that the development of medullary thymic epithelial cells (mTECs) depends on NF-κB associated signaling, it remained unclear whether other signaling pathways are also involved. In this context, it had been reported that conditional deletion of Stat3 alleles in TECs using cytokeratin-5 (CK5) promoter controlled Cre expression results in a profound impairment in TEC development. However, a detailed analysis of phenotypes in mTECs remained unstudied. In the present study, we show that thymic medullary regions remain as small islets when Stat3 is conditionally deleted in thymic epithelial cells, while they normal fuse to form continuous structures during postnatal development. Furthermore, we identified EGF-R mediated signal to be placed upstream of Stat3 activation, as its ablation phenocopied the loss of Stat3 expression in TECs. Thus, the present study revealed that Stat3 is required for the postnatal development of medullary regions.

## Introduction

Throughout life, the thymus serves as a primary lymphoid organ for the production of T cells. The thymic environment comprises two distinct domains, the cortex and the medulla, which are mainly composed of thymic epithelial cells (TECs) organized in a three dimensional architecture [1,2]. The cortex serves as the site for early and intermediate T cell development, including commitment of progenitors to the T cell lineage, and the proliferation and positive selection of developing thymocytes [3,4,5]. Recently, it has been shown that negative selection also takes place in the cortex [6,7], which is thought to be induced by dendritic cells [8,9]. The medulla supports the final steps in T cell development, including the deletion of T cells reactive to a tissue-restricted self-antigens (TRA), typically but not exclusively expressed by medullary TEC (mTEC) via a yet incompletely understood mechanism of promiscuous gene expression [10–13]. Expression of some TRA depends on the AutoImmune REgulator (AIRE), a nuclear factor present in a subpopulation of mature mTEC [14], that facilitates a very broad, context-dependent, probabilistic, and noisy transcription. Loss of AIRE expression results in an incomplete representation of TRA in mTEC and, consequently, in an aberrant T cell antigen receptor (TCR) repertoire comprising self-reactive T cells able to elicit autoimmunity [14].

Both cortical TECs (cTECs) and medullary TECs (mTECs) arise during fetal development from a common epithelial progenitor derived from third pharyngeal pouch endoderm [15,16,17]. In the mouse, the primary segregation into cortical and medullary domains occurs from 13 days post coitum (dpc) onwards [18,19,20]. Further development of cortex occurs along with the differentiation and expansion of thymocytes from CD4^-^CD8^-^ (double negative; DN) stage to CD4^+^CD8^+^ (double positive; DP) stage [21]. Whereas the formation of the medullary anlage and the initial differentiation of mTECs is initiated and proceeds during the fetal period, the realization of the medullary architecture is only initiated around birth and parallels the emergence of mature CD4 and CD8 single positive (SP) thymocytes [22]. The size of the thymus reaches its maximum early in adulthood and involutes progressively thereafter [23]. Along with the involution, thymic output decreases, leading to the defective function of peripheral T cells [24].

The population of mTECs can be distinguished on the basis of phenotypic markers into separate subpopulations, which seemingly represent consecutive developmental stages [25–29]. mTECs have a half life of 2 to 3 weeks and are therefore continuously replaced from a precursor pool of so far not further characterized epithelia [25,30–33].

Growth and maturation of TECs are critically controlled by developing thymocytes via a process of physical and functional interactions, a phenomenon referred to as “thymic cross-talk”[22,34,35]. Whereas the development of cTECs occurs in response to the developmental progress of thymocytes from DN to DP stage [18], the expansion and differentiation of mTECs occurs as a consequence of signals provided by SP thymocytes [22,36]. Previous studies have revealed that IkB kinase (IKK) RelB, NF-κB inducing kinase (NIK), and TRAF6 and the upstream positioned cell surface molecules RANK, CD40 and LTβR are required for physiological mTEC development [37–46].

Whether additional signaling pathways other than non-canonical NF-κB signals control mTEC development is still incompletely understood. It was previously reported that Signal transducer and activator of transcription 3 (Stat3) signaling is critical for postnatal TEC maintenance, as its conditional inactivation, using Krt5.Cre-mediated recombination was shown to cause severe thymic hypoplasia as early as 5 weeks of age [47]. Stat3 is one of a family of cytoplasmic proteins that participate in normal cellular responses to cytokines, growth factors and other cell extrinsic influences such as ionizing radiation [48]. The study also implicated that the underlining mechanism of thymic hypoplasia, is linked to the cTEC compartment.

In the present study, we wished to further delineate the upstream mechanisms responsible for the activation of Stat3 signaling in TECs. To this end, and in reference to previously published data [47], we first generated mice with a TEC-targeted loss of Stat3 gene function employing Foxn1-Cre mice as a tissue-specific driver for gene deletion [49]. Contrary to the reported findings using K5-Cre mice to delete Stat3, Foxn1-Cre-driven inactivation of the Stat3 locus in TEC resulted in juvenile and adult mice in a normal sized thymus with a reduced medulla but a normal cortex. These results thus demonstrate that Stat3 is required for the maintenance of mTECs, but dispensable for the growth and the up-keep of cTECs during postnatal life contrary to what had previously been concluded. Moreover, our studies revealed that EGF-R operates upstream of Stat3 as mice conditionally deficient for EGF-R in TEC displayed a thymic stromal phenotype identical to that of Stat3 deficient animals.

## Results

### Reduced size of thymic medullary regions in Stat3 deficient mice

To study the role of Stat3-mediated signals in thymic epithelial development and function, mice with a loss of Stat3 expression in TEC were generated using Foxn1-driven Cre recombination [49] of the conditional Stat3 locus (Foxn1-Cre::Stat3-f/f; hereafter designated Foxn1-Stat3-CKO mice). Foxn1 is a cell-autonomous master regulator expressed in all TEC subpopulations [50,51] and critically important for differentiation and growth [50,52,53]. We confirmed that Cre-Lox system works well in both cTECs and mTECs (S1 Fig). We also checked whether expression of Cre driven by Foxn1 exerts any toxicity for thymic development, and found no abnormality on thymic architecture in Foxn1-Cre:Stat3-f/+ mice compared with that in Stat3-f/f mice (S2 Fig).

**Fig 1:**
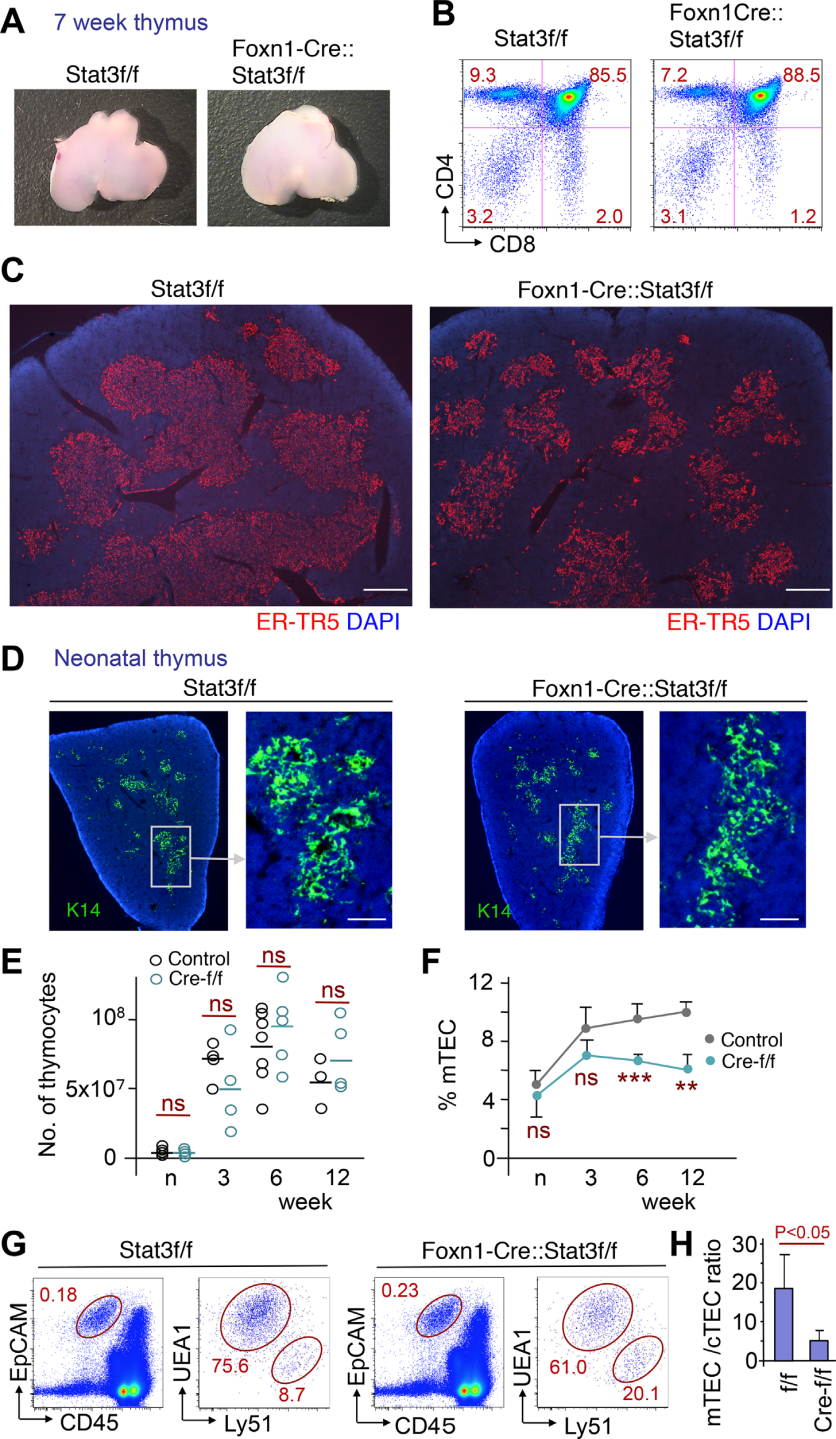
Medullary regions are severely affected in Foxn1-Stat3-CKO mice. (A) Macroscopy of the thymus derived from Stat3-flox/flox (Stat3f/f) and Foxn1Cre::Stat3f/f mice at 7 weeks of age. (B) Flowcytometric profiles of developing thymocytes derived from Stat3f/f and Foxn1Cre::Stat3f/f mice, at 7 weeks of age. (C) Cryostat sections of thymic tissue from Stat3f/f and Foxn1Cre::Stat3f/f mice (7 weeks of age) were stained with the mTEC specific antibody, ER-TR5 (red) and counterstained with DAPI (blue). Scale bars: 400 μm. (D) Cryostat sections of neonatal thymic tissue from Stat3f/f and Foxn1Cre::Stat3f/f mice were stained with antibody directed to K14 (green) and counterstained with DAPI (blue) Scale bars: 100 μm. (E) Total thymic cellularity of control (containing Cre-f/+ and f/f) and Foxn1Cre::Stat3f/f (Cre-f/f) mice at indicated ages. Bar stands for the average value of each experimental group. ns denotes a non-significant difference (P>0.1) in Student’s t test. (F) Changes in the proportional area of medullary regions in thymus tissue sections of control (containing Cre-f/+ and f/f, n = 8, 6, 7, 3 for neo, 3, 6, 12 week, respectively) and Foxn1Cre::Stat3f/f (Cre-f/f, n = 5, 4, 6, 5 for neo, 3, 6, 12 week, respectively) mice in the first 12 weeks of life. The area occupied by mTECs in thymus was quantitatively measured in sections stained with K14 antibody using Axiovision4 software (Carl Zeiss). Error bar stands for the standard deviation. ns denotes a non-significant difference (P>0.1) in Student’s t test. **;P<0.005, ***;P<0.0005. (G) Representative flow cytometric profiles showing frequencies of major TEC populations from 12 weeks old Stat3f/f and Foxn1Cre::Stat3f/f mice. EpCAM^+^CD45^-^ fraction represents whole TEC population, and UEA1 vs Ly51 profile was displayed for the cells gated on EpCAM^+^CD45^-^ fraction, where UEA1^high^Ly51^low^ and UEA1^low^Ly51^high^ fraction were defined as mTEC and cTEC population, respectively. (H) Ratio of mTEC vs cTECs in flow cytometric analysis of control (containing Cre-f/+ and f/f, n = 4) and Foxn1Cre::Stat3f/f mice (n = 5) at 12 weeks of age is shown.

To our great surprise and contrary to findings in mice where Stat3 was deleted in TEC using the expression of Cre under the control of the Keratin 5 promoter (K5-Cre:: Stat3-f/f, designated K5-Stat3-CKO) [47], changes in the overall size of the thymus and thymocyte differentiation as assessed by CD4 and CD8 cell surface expression were not observed in Foxn1-Stat3-CKO mice (Fig 1A and 1B). However, the immunohistological analyses of thymus tissue sections revealed that the TEC targeted absence of Stat3 expression significantly reduced the size of the medulla, resulted in a fragmentation of its island architecture and led to a decrease in the number of mTEC (Fig 1C). These striking changes were only apparent in Foxn1-Stat3-CKO animals 6 weeks and older (Fig 1D, 1E and 1F**)** and strongly imply a role for Stat3 in the physiological development of thymic medullary regions, where medullary islets are fused to form continuous architecture during postnatal period [54]. We confirmed this decrease in mTEC number by flow cytometric analysis of thymus from control and Foxn1-Stat3-CKO mice at 12 weeks of age (Fig 1H**)**. Very similar results was seen in the experiments using a different strain of Foxn1-Cre mice [55], where ratio of mTECs was reduced in thymus of Foxn1-Stat3-CKO mice (S3 Fig).

### mTEC differentiation is not impaired in the absence of Stat3

**Fig 2:**
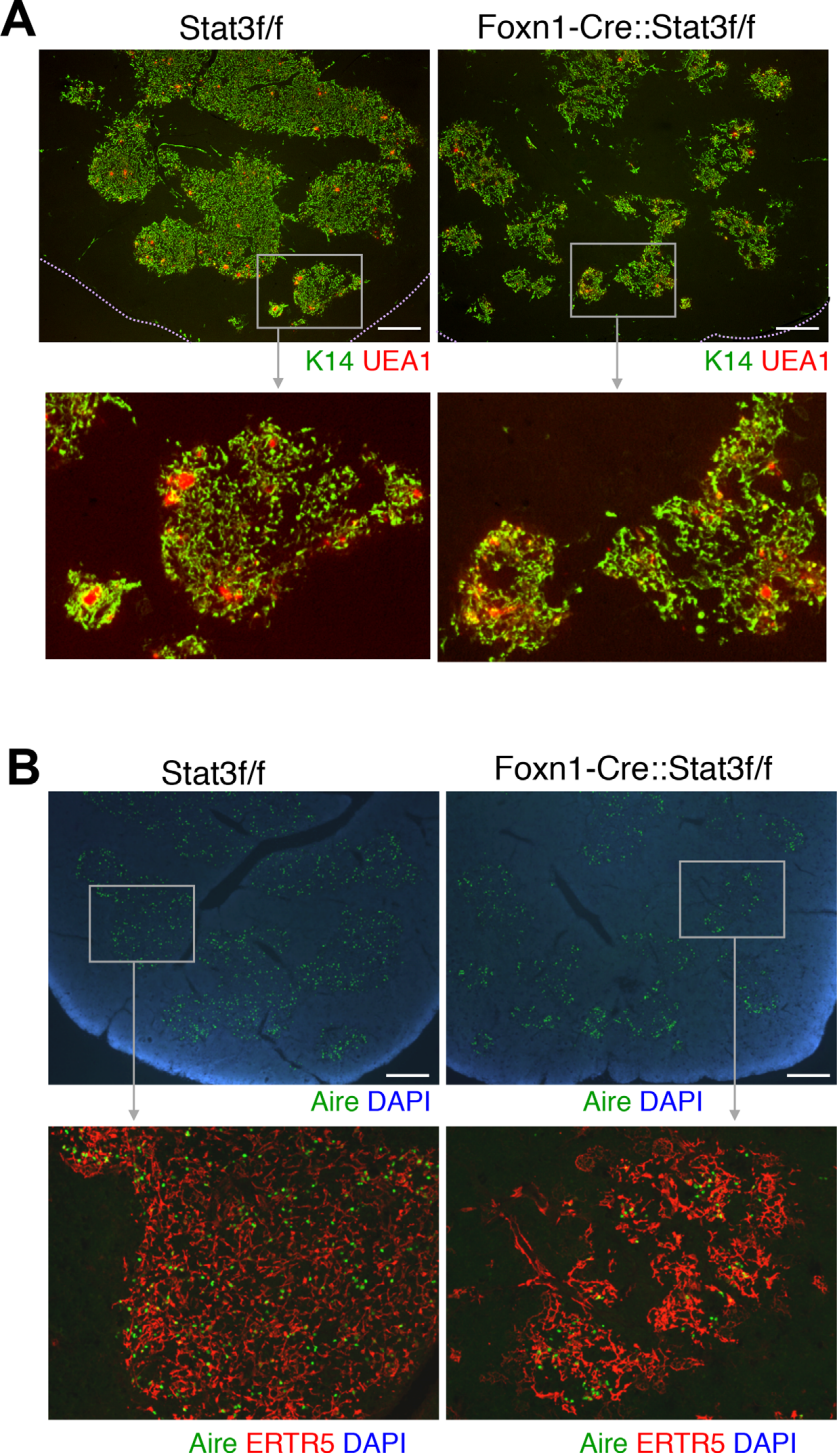
Maturation of mTECs was not affected in Foxn1-Stat3-CKO. Cryostat sections of the thymus were stained with antibodies directed to K14 (green) and UEA1 (red) (A), and with antibodies to ERTR5 (red) and AIRE (green) (B). Sections were counterstained with DAPI (blue). Scale bars: 400 μm.

We next established whether Stat3–deficient mTECs are developmentally impaired. For this purpose, thymus sections of 6 week old Stat3f/f and Foxn1-Stat3-CKO mice were analyzed for the expression of K14, a pan-mTEC marker, and UEA-1, a marker characteristic for the immunohistological identification of differentiated mTECs. The loss of Stat3 expression in mTEC did not affect their UEA1 staining pattern (Fig 2A**)**. These results indicate that the maturation of mTECs is not affected by the absence of Stat3. Moreover, AIRE expressing mTECs were seen in a similar manner among mTEC of Foxn1-Stat3-CKO mice (Fig 2B) excluding the possibility that Stat3-mediated signals are required for the expression of Aire. In addition, number of thymic regulatory T cells as well as splenic ones was found to be intact. Collectively, these results showed that Stat3 is indispensible for the postnatal growth of mTECs and maintenance of mTEC cellularity, while dispensable for functional maturation of mTECs.

### Stat3-mediated signaling is dispensable in cTEC

**Fig 3:**
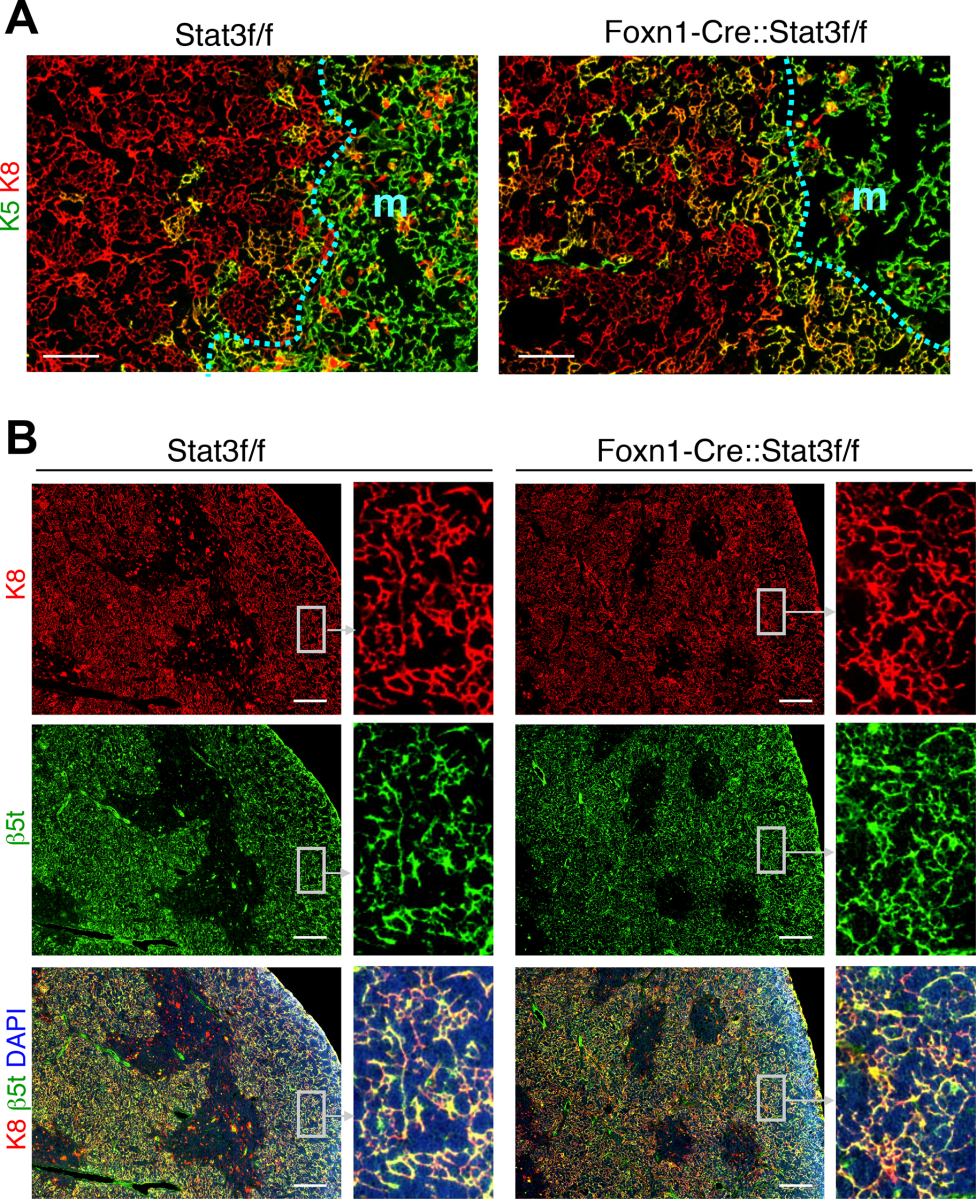
Normal differentiation of cTECs in Foxn1-Stat3-CKO mice. (A) Cryostat sections of the thymus were stained with antibodies directed to K8 (red) and to K5 (green). The dotted blue line indicates the cortico-medullary junction, separating cortex (c) from the medulla (m). Scale bars: 100 μm. (B) Cryostat sections of the thymus were stained with antibodies directed to K8 (red) and to β5t (green). Sections were counterstained with DAPI (blue). Scale bars: 200 μm.

In light of the importance of Stat3-mediated signals for mTEC growth, we next investigated whether cTEC development and maintenance in Foxn1-Stat3-CKO also required Stat3 for their expansion since Stat3 was robustly expressed in these cells [13]. Indeed, an earlier study analyzing K5-Stat3-CKO mice 5 weeks and older had reported a significant loss of cTEC with the remaining cTEC organised parallel to the thymic capsule and revealing a thymic nurse cell-like phenotype [47]. In sharp contrast, Foxn1-Stat3-CKO mice displayed a thymic cortex of normal size with a regular 3-D architecture of cTEC that was in comparison to wild type mice indistinguishable (Fig 3A). The vast majority of cTEC were stained positively for the cTEC-lineage marker β5t [56] (Fig 3B). Thus, the analysis of Foxn1-Stat3-CKO mice suggested that Stat3 and its downstream signaling pathways were dispensable for cTEC differentiation, homeostatic maintenance, organization and function as highlighted by a regular thymus cellularity, cortical histology and intrathymic T cell differentiation (Fig 1A, 1B and 1E).

**Fig 4:**
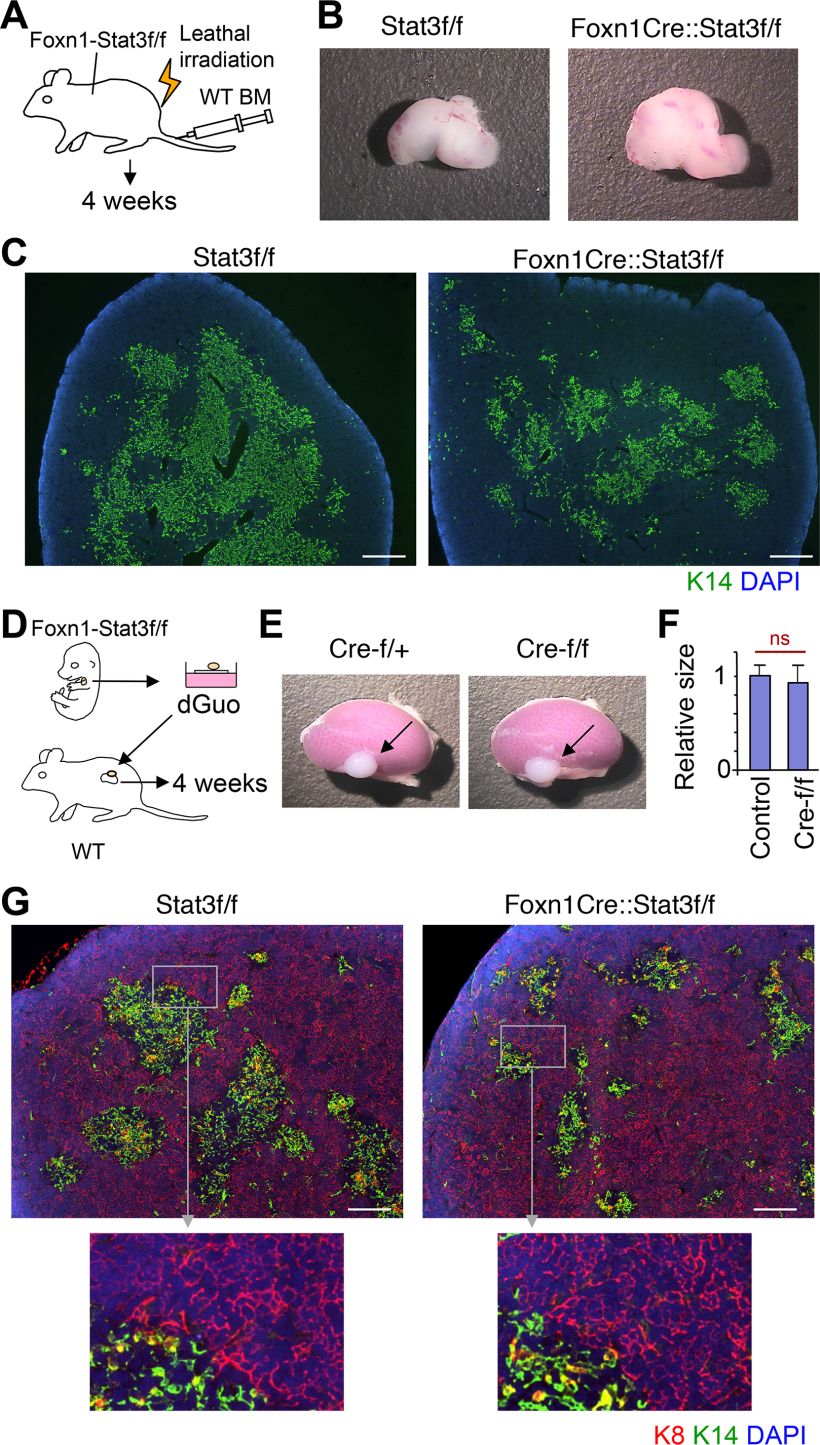
Regenerative potential of cTECs was not affected in Foxn1-Stat3-CKO mice. (A) Experimental procedure for (B) and (C). Foxn1-Cre::Stat3-f/f mice were lethally irradiated and then rescued by bone marrow transplantation from wild type mice. After 4 weeks, mice were sacrificed and thymic tissue was examined. (B) Macroscopy of the thymus 4 weeks after hematopoietic stem cell transplantation. (C) Cryostat sections of the thymus were stained with anti-K14 antibody (green) and counterstained with DAPI (blue). Scale bars: 400 μm. (D) Experimental design for data presented in panels (E) and (F). Foxn1-Cre::Stat3-f/f fetal thymic lobes (15 dpc) were cultured in vitro for 6 days in the presence of deoxyguanosine and subsequently transplanted under kidney capsule of wild type mice. After 4 weeks, mice were sacrificed and thymic grafts were examined. (E) Gross anatomical analysis of the thymic grafts 4 weeks after grafting into C57BL/6 wild type recipients. (F) Comparison of maximum cross-section area of thymic grafts of control (containing Cre-f/+ and f/f, n = 4) and Foxn1Cre::Stat3f/f mice (n = 4). (G) Cryostat sections of thymic grafts were stained with anti-K8 (red) and anti-K14 antibody (green). Sections were counterstained with DAPI (blue). Scale bars: 200 μm.

Experiments reported by Sano et al [47] had shown that the thymic environment of their K5-Stat3-CKO mice could not recover fully after ionizing irradiation (IR), and hematopoietic stem cell rescue, suggesting a role for Stat3 in the regenerative response to radiation. We therefore extended these studies to Foxn1-Stat3-CKO and the control Stat3f/f mice (Fig 4A**)**. In contrast to K5-Stat3-CKO, the thymic architecture of lethally irradiated and transplanted Foxn1-Stat3-CKO mice and their controls recovered normally and adopted within 4 weeks a phenotype identical that observed in untreated animals (Fig 4B and 4C). To further test the resilience of Stat3-deficient TEC to other noxious stimuli, we next exposed day 15 (15dpc) fetal thymic (FT) lobes isolated from either Foxn1-Stat3-CKO or Stat3f/f mice to deoxyguanosine (dGuo). This *in vitro* treatment depletes hematopoietic cells, abrogates thymic cross-talk and consequently impairs TEC function [57]. After 6 days in culture, dGuo treated FT lobes were grafted under the kidney capsule of syngeneic wild type mice (Fig 4D). The gross anatomical analysis and measurement of maximum cross-section area of the grafts 4 weeks later failed to demonstrate a difference in size between the transplanted K5-Stat3-CKO and control thymic tissues (Fig 4E and 4F). A histological examination revealed—as expected–a smaller medulla separated into minimal islands but a regular sized cortex with a typical TEC architecture (Fig 4G). These experiments therefore demonstrated that Stat3 is not required for regeneration following IR and the loss of extended thymic cross-talk.

### K5-Cre::Stat3 f/f and Foxn1-Cre: Stat3f/f mice kept in the same mouse colony do not differ in their thymic phenotype

**Fig 5:**
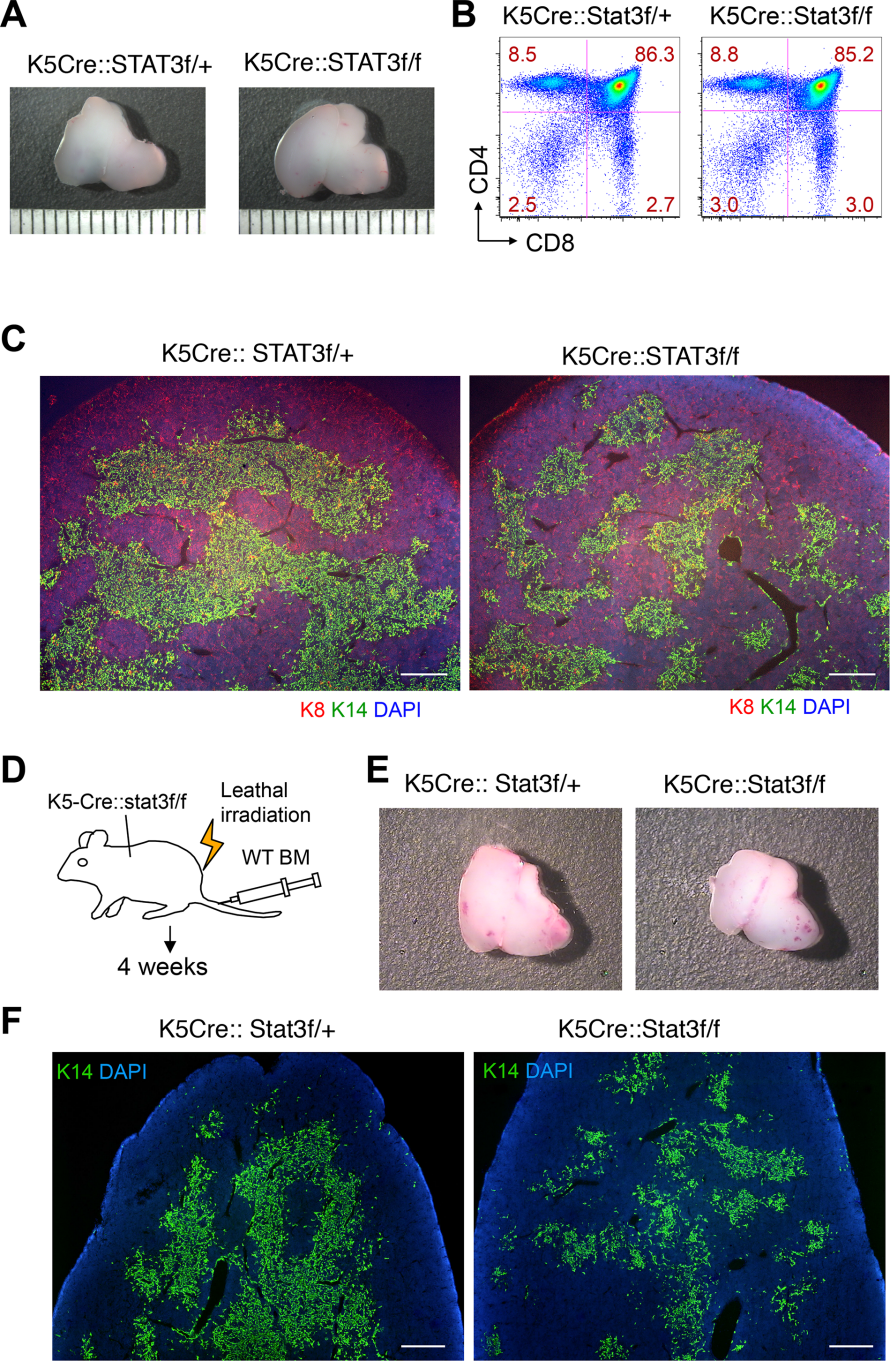
Medullary regions are severely affected while cTECs were normally regenerated in K5-Cre-Stat3-CKO mice. (A) Gross anatomical analysis of the thymus derived from 7 week old K5Cre::Stat3f/+ and K5Cre::Stat3f/f mice. Scale bars: 1 mm. (B) CD4 and CD8 cell surface expression on thymocytes derived from 7 week old Stat3f/f and K5Cre::Stat3f/f mice. (C) Cryostat sections of thymic tissues derived from either K5Cre::Stat3f/+ or K5Cre::Stat3f/f mice at 7 weeks of age. The tissues were stained with anti-K8 (red) and anti-K14 (green) antibody and counterstained with DAPI (blue). Scale bars: 400 μm. (D) Experimental design for data presented in panels (E) and (F). K5-Cre::Stat3-f/f mice were lethally irradiated and then rescued by hematopoietic stem cell transplantation (wild type C57BL/6). After 4 weeks, mice were sacrificed and the thymus was examined. (E) Gross anatomical analysis of the grafted thymus 4 weeks after hematopoietic stem cell transplantation. (F) Cryostat sections of the thymus were stained with anti-K14 antibody (green) and counterstained with DAPI (blue). Scale bars: 400 μm.

Differences in the pattern of Cre expression due to the use of different promoters, variance in the genetic background of the animals analyzed, or dissimilarities in colony conditions could account for the remarkable phenotypic and regenerative differences observed between Foxn1-Stat3-CKO and K5-Stat3-CKO mice. To address these issue, we next established a K5-Stat3-CKO colony in the same animal facility where Foxn1-Stat3-CKO mice had already been housed. For this purpose, we used K5-Cre mouse line [58], which is the same one as used in the study by Sano et al [58]. Surprisingly, six week old, K5-Stat3-CKO mice displayed a phenotype identical to that of Foxn1-Stat3-CKO mice. Specifically, differences in thymus size (Fig 5A), intrathymic T cell maturation (Fig 5B), or epithelial organization (Fig 5C) could not be observed. Moreover, the regenerative responses of the thymic stromal compartment to irradiation and dGuo treatment were identical for both mouse strains (Figs 4, 5D, 5E, 5F and S4). We therefore concluded that genetic differences could not account for the phenotypic disparities reported here and those described by Sano et al [47].

### Normal intrathymic T cell development in aged Foxn1-Stat3-CKO mice despite medullary hypoplasia

Because a reliance on Stat3 had been particularly noticeable in older mice [47], we extended our histological and flow cytometric analysis of the thymic microenvironment to 20 months old Foxn1-Stat3-CKO and Stat3fl/fl control mice. The cortex displayed in both mouse strains an age-appropriate involution (S5A Fig). The medulla had fused to form a contiguous and prominent compartment in control mice whereas the medulla of older Foxn1-Stat3-CKO remained restricted in size and continued to be composed of separate small islands **(**S5B Fig). Despite these structural differences, the flow cytometric profiles for CD4 and CD8 expression on thymocytes (S5C Fig) and the number of T cells newly emigrated from the thymus to the periphery (as measured by the quantification of T cell receptor excision circles, TREC) were comparable for Foxn1-Stat3-CKO and Stat3fl/fl mice (S5D Fig). A normal thymic out-put was furthermore reflected in a regular proportion of naïve and memory T cells within each of the CD4^+^ and CD8^+^ T cell populations of old Foxn1-Stat3-CKO and control mice (S5E Fig). Finally, the frequency of regulatory T cells was normal both in the thymus and periphery of Foxn1-Stat3-CKO mice and comparable to that of age-matched control animals (S5F and S5G Fig). Recent study has shown that regulatory T cells are prominently reduced in RelB deficient thymus which completely lack functional medulla [59]. In Foxn1-Stat3-CKO thymus, it is probable that the small medullary regions may be functional enough to maintain normal number of regulatory T cells. Collectively, our data obtained in very old mice indicated that a lack in Stat3 expression in thymic epithelia did not impact on the production, exit and maintenance of T cells.

### EGF-R mediated signaling is upstream of Stat3 activation and involved in the postnatal maintenance of the thymic medulla

**Fig 6:**
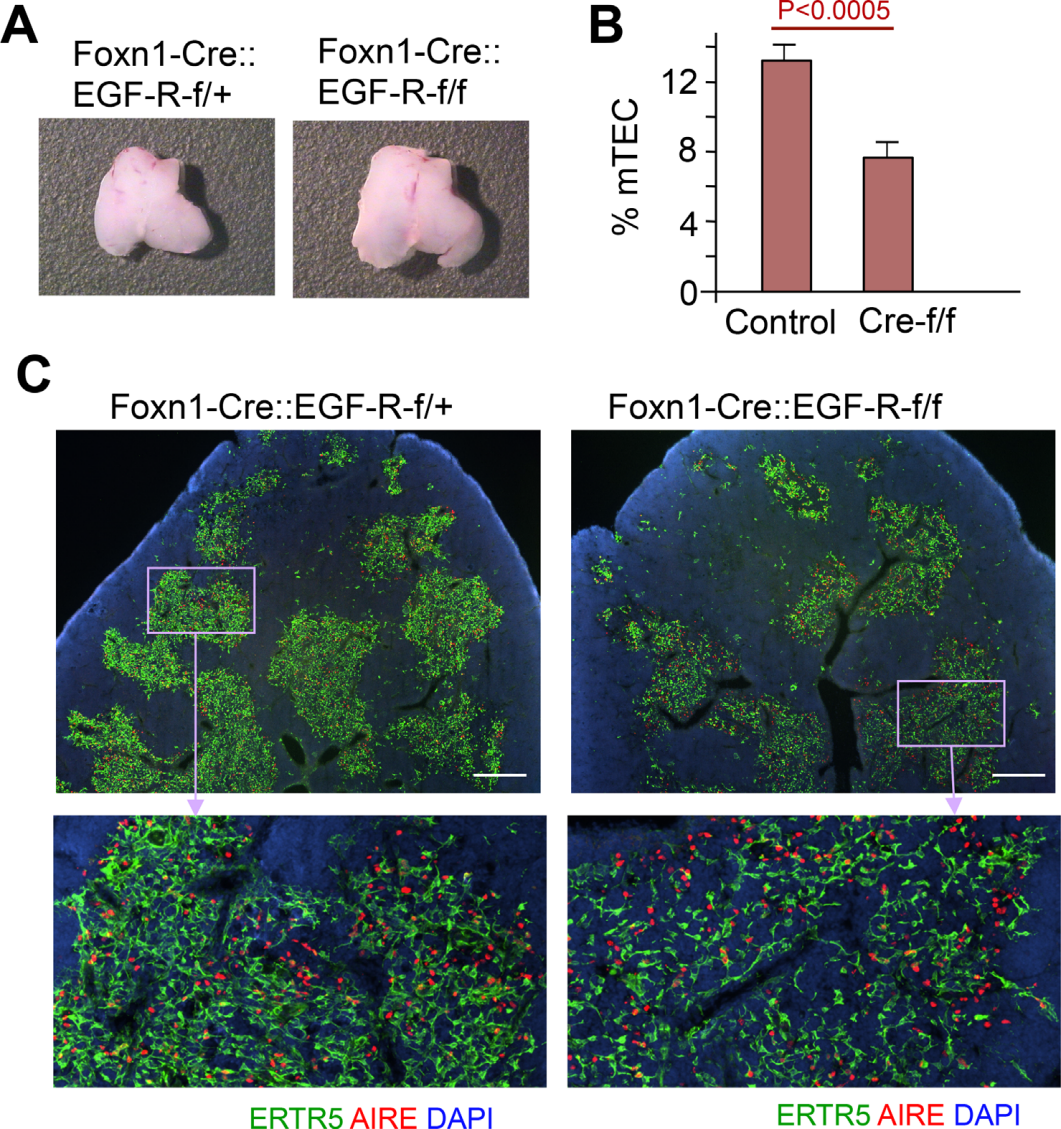
mTECs are reduced in Foxn1-EGF-R-CKO mice. (A) Gross anatomical analysis of the thymus derived from Foxn1Cre::EGF-Rf/+ mice (control) and Foxn1Cre::EGF-Rf/f mice at 6 weeks of age. (B) Quantitative analysis for the proportion of medullary regions in thymus of control (containing Cre-f/+ and f/f, n = 4) and mutant (Cre-f/f, n = 5) mice. (C) Cryostat sections of the thymus were stained with ER-TR5 antibody (green) and anti-AIRE antibody (red). Sections were counterstained with DAPI (blue). Scale bars: 400 μm.

Since Stat3 is indispensible for the post-natal growth of mTEC, we next investigated receptors that transduce their activation signals via phosphorylation at tyrosine 705 of Stat3. Various cytokines and growth factors have been demonstrated in different tissues to mediate Stat3 activation, including hepatocyte growth factor (HGF, a.k.a. Met) and epidermal growth factor (EGF) [60]. Since the corresponding receptors, HGF receptor (HGF-R) and EGF receptor (EGF-R), are expressed on both cTECs and mTECs (S6A Fig), we sought to delete their expression exclusively in TEC. For this purpose, we crossed mice with loxP-flanked *met* and *egfr* alleles, respectively, to Foxn1-Cre mice. Animals with a lack of *met* expression in TEC did not display any changes in cellularity, phenotype or architectural composition when compared to wild type controls irrespective of their age (S6B Fig). In contrast, the thymus of adult Foxn1-EGF-R-CKO mice, although normal in size (Fig 6A), demonstrated in comparison to wild type, age-matched controls a thymic medulla marked by small independent islands with fewer ER-TR5^+^ cells forming a less dense stromal meshwork (Fig 6B and 6C). The expression of AIRE was, however, unaffected by the absence of EGF-R expression (Fig 6C). This phenotype well overlapped with that of Foxn1-Stat3-CKO mice, although fragmentation of medullary islets seemed a bit milder. In addition, the phenotype of Foxn1-EGF-R-CKO thymus was not further altered by an additional loss of HGF-R expression (S6B Fig). These findings identify EGF-R as the relevant signaling node upstream of Stat3 phosphorylation in TEC and exclude signals downstream of HGF-R to contribute to the growth and organization of the thymic medulla.

## Discussion

Our study demonstrates that Stat3-mediated signaling is indispensible for the postnatal development of a thymic medulla, failure of which leads to a limited number of separate medullary islands. Among the several upstream transduction pathways activating Stat3, the stimulation of the EGF-R plays an essential role in the maintenance of the medulla throughout life. EGF-R or alternative signaling pathways reliant on Stat3 activation are, however, neither used for the growth nor essential for the function of cortical TEC. Our findings therefore uncover molecular and temporal differences in the up-keep of the two separate TEC lineages and challenge the previously described role for Stat3 in TEC maintenance/development [47].

A large number of cytokines and growth factors, including EGF and HGF, and their corresponding receptors, have been position upstream of the Stat3 activation. When bound to their cognate receptors, they transiently activate Stat3, which in turn modulates the transcription of responsive genes involved in various cellular functions [60–63]. Using TEC specific loss-of-function mutants, we now directly demonstrate that EGFR but not HGFR is required in the biology of post-natal TEC. Stat3 controls the transcription of several target genes including the neuroendocrine hormone insulin-like growth factor 1 (IGF-1) [64]. IGF-1 which is robustly expressed in medullary TEC but barely detectable in cortical TEC [65] has been shown to predominantly effect thymic function through its paracrine/autocrine effects on TEC numbers and function [66]. Transcripts for the receptor of IGF-1, IGF-R, are differentially expressed between these two separate anatomical compartments with cTEC displaying higher copy numbers [67]. A differential dependence of the individual TEC subpopulations to IGF-1-mediated autocrine activation may thus be a possible explanation for the presence of a medullary but the absence of cortical phenotype in the Foxn1-Cre-Stat3 mice presented.

The phenotype observed in Foxn1-Cre-Stat3 and the conclusions drawn from our results contrast the findings previously communicated in another report that analyzed post-pubertal mice in which Stat3 was deleted in K5 positive TEC [47]. Namely, in the original report the K5-Stat3-CKO mice developed severe thymic hypoplasia with alterations in the cortical TEC architecture that coincided with a loss of thymocytes whereas medullary TEC displayed a relatively normal appearance. The striking variance in the cortical phenotypes observed between the K5-Stat3-CKO mice originally described and the K5-Stat3-CKO animals reported here can obviously not be accounted for by differences in the gene targeting strategy. Therefore, one possible explanation for the apparent discrepancy between the studies may be due to the difference in genetic background of the specific animal colonies compared and/or differences in the animal housing. While our strains, whether housed in Japan or in Israel, were on a pure C57BL/6 background, the mice used in the original study could be on the way of backcrossing from mixed 129-C57BL/6 status to pure C57BL/6. Another possible explanation can be suggested by the finding that the two mouse colonies differed also in their skin phenotype. Although the initial morphogenesis of the skin appeared normal in K5-Stat3-CKO mice reported earlier [68], older animals spontaneously developed skin ulcers and alopecia and demonstrated impaired wound healing. Local inflammatory changes due to a loss in the regular barrier function of the skin in K5-Stat3-CKO could lead to systemic consequences including high serum corticosterol levels that in turn cause thymus cortical hypoplasia. Indeed, mice treated with dexamethasone display alterations in the composition and organization of the thymic microenvironment that are comparable to those observed in the K5-Stat3-CKO mice previously reported [47,69]. Our own experiments in which both types of Stat3 conditionally-deficient mice fully recover from radiation- or deoxyguanosine-mediated damage provide then further evidence that the severe skin lesions very likely contribute to the observed thymic phenotype in K5-Stat3-CKO as observed by Sano and colleagues and thus constitute a secondary phenomenon.

In aggregate, our results obtained using several mouse strains in independent laboratories demonstrate that Stat3-mediated signaling input from EGF-R determines in post-natal mice the growth and architectural organization of mTEC. In contrast, Stat3-mediated signaling is dispensable for the biology of cTEC. These findings are in keeping with the results reported by Lomada *et al*. (co-submitted manuscript) but cannot confirm the conclusion of the previously reported study by Sano *et al*. [47]. While the molecular mechanisms accounting for the observed differences remain to be elucidated, experimental evidence from our studies using independently generated mouse strains housed in separate animal facilities each with individual environmental conditions would firmly conclude that Stat3-mediated signals are essential for medullary but superfluous for cortical thymic epithelia.

## Materials and Methods

### Mice

C57BL/6 (B6) mice were purchased from CLEA Japan Inc (Tokyo, Japan). Foxn1-Cre BAC transgenic mice [49] were maintained in our animal facility. A different strain of Foxn1-Cre mice, based on IRES-Cre knockin into the Foxn1 locus [55], were maintained and analyzed at the Weizmann Institute and were a kind gift of Prof. Nancy Manley. Stat3-flox/flox mice were donated by Prof. Shizuo Akira [70], K5-Cre mice were donated by Prof. Junji Takeda [58]. EGFR-flox/flox mice and Met-flox/flox mice were donated by Prof. Maria Sibilia [71] and Prof. Carmen Birchmeier [72]. Stat3-flox/flox and K5-Cre mice maintained and analyzed at the Weizmann Institute were a kind gift of Prof. Shizuo Akira and Prof. Dennis Roop, respectively. Embryos at the indicated stages of gestation were obtained from time-mated pregnant mice. The day of detecting a vaginal plug was designated as 0 days post conception (dpc).

### Antibodies

The following antibodies were used for flow cytometric studies: anti-CD8 (53–6.7), anti-CD4 (H129.19), anti-CD3ε (145-2C11, 500A2), anti-CD62L (MEL-14), anti-CD44 (IM7), and anti-CD25 (PC61), anti-CD19 (1D3), Mac-1 (M1/70), γδ TCR (UC7-13D5) (all purchased from BD PharMingen, San Jose, CA). For immunohistochemistry, the following antibodies were used: Rabbit anti-cytokeratin 14 (K14; rabbit, COVANCE, Princeton, NJ), rabbit anti-K5 (rabbit, COVANCE, Princeton, NJ), and biotinylated mouse anti-K8 (PROGEN, Heidelberg, Germany), biotinylated UEA-1 (VECTOR LABORATORIES, Burlingame, CA), rabbit anti-β5t (MBL, Nagoya, Japan), ERTR5 [73]. Polyclonal anti-Aire antibody was a kind gift from M. Matsumoto (Tokushima Univ.). All secondary reagents for immunohistochemistry were purchased from Molecular Probes (Carlsbad, CA): Alexa Fluor488 donkey anti-rabbit IgG (H+L) conjugate, Alexa Fluor488 goat anti-rabbit IgG (H+L) conjugate, Alexa Fluor488 streptavidin conjugate, Alexa Fluor546 goat anti-rabbit IgG (H+L) conjugate and Alexa Fluor546 streptavidin conjugate.

### Regulatory T-cell staining

Anti-FoxP3 (FJK-16S) antibody was from eBioscience, and intracellular FoxP3 staining was performed according to the manufacturer’s instruction (eBioscience, San Diego, CA).

### Quantification of mouse T-cell receptor (TCR)δ signal joint excision circles (mTREC)

To obtain splenic CD4 single positive (SP) and CD8SP T cells, red blood cells were lysed using a RED BLOOD CELL LYSING BUFFER (Sigma, St. Louis, MO) followed by the depletion of CD19, Mac1 and γδT cells with magnetic beads. CD4SP cells and CD8SP cells were sorted by a FACS AriaⅢ (Becton Deckinson). Sorted cells were lysed in ProteinaseK (Sigma, St. Louis, MO) to prepare genomic DNA. An mTREC were quantified as previously described [74]. An mTREC standard for the real-time PCR was generated by PCR cloning of a 586-base-pair fragment of mTREC DNA from C57BL/6 mouse thymus genomic DNA into pCR4Blunt-TOPO (Invitrogen, Carlsbad, CA). An mTREC of splenic CD4SP and CD8SP cells were amplified and quantified by ABI StepOnePlus using Power SYBR Green PCR Master Mix (Applied Biosystems). Primers used to detect mTREC were as follows: Forward primer: 5’ -TCATTGCCTTTGAACCAAGC- 3’, Reverse primer: 5’–CACAGCAGCTGTGGGTTTATG- 3’

### Fetal thymic organ culture and reconstitution experiments

To deplete thymocytes, fetal thymic (FT) lobes 15 dpc embryos were cultured for 6 days on polycarbonate filters (pore size 8.0 μm) (Nucleopore Co., Pleasanton, CA) in the presence of RPMI 1640 medium supplemented with 10% FCS and 1.35 mM dGuo (Nacalai Tesque, Kyoto, Japan) [75]. Where indicated, single dGuo-treated lobes were grafted under the kidney capsule of recipient mice and analyzed 1 month later.

### Hematopoietic stem cell transplantation

Bone marrow cells from C57BL/6 mouse were transplanted into lethally irradiated Foxn1Cre::Stat3flox/flox mice or K5Cre::Stat3flox/flox mice (10^7^ bone marrow cells per mouse). The chimeric mice were analyzed 1 month later.

### Immunohistochemistry

All thymic lobes were embedded in OCT compound (Sakura Fine Tek, Tokyo, Japan) in Leica Histomolds (Leica Microsystems, Wetzlar, Germany) and snap-frozen in liquid nitrogen. Serial sections (5 μm) tissue sections were cut from frozen blocks using a Leica CM3050S cryostat and were subsequently mounted onto MAS-coated slides (Matsunami Glass Ind. LTD, Osaka, Japan). After acetone fixation for a few seconds, sections were incubated with primary antibodies (1hr, room temperature), washed 5 times with PBS/0.05% Tween and then incubated with secondary reagents (30min, room temperature). Nuclei were counterstained with DAPI (Molecular Probes).

### Flow cytometric analysis of TECs

Preparation of thymic epithelial cells was performed as previously described [76]. Thymic tissue were cut into small pieces by forceps and placed into 15 mL tube containing 2mL of RPMI-1640 (Sigma) + 1% FCS. After pipetting and settling for 2 min, the supernatant were discarded. This was repeated several times. RPMI-1640 + 1% FCS containing 0.5 U/mL Liberase TM (Sigma-Aldrich) and 0.02% (w/v) DNaseⅠ (Roche) were added to remaining thymic fragments and incubated at 37°C for 12 min. After settling for 2 min, the supernatant were collected into 15 mL tube and suspended with PBS (-) + 1% FCS + 5 mM EDTA. This step was repeated twice. After washing cells, they were passed through mesh. Single cell suspension was stained with CD45 (clone 30-F11, eBioscience), EpCAM (clone G8.8, eBioscience), Ly51 (clone 6C3, BD Pharmingen) and UEA-1 (Vector Laboratories) and sorted by using FACSAriaⅢ (Becton Dickinson).

### Genotyping of sorted cells

PCR experiment of sorted cells was performed to confirm the deletion of floxed allele.

The following primers were used (a-b; germline, b-c; deleted configuration).

- AGCAGCTGACAACGCTGGCTGAGAAGCT
- TTGCTGCTCTCGCTGAAGCGCAGTAGG
- GATTTGAGTCCAGGGATCCATAACTTCG

### Ethics statement

Animal care and experiments were conducted according to the guidelines established by the RIKEN Yokohama experiments committee (approval number is: K24-020). Pentobarbital and CO_2_ gas was used for anesthesia, and for euthanasia of mice, respectively.

## Supporting Information

S1 Fig Stat3 is efficiently deleted in both mTECs and cTECs. cTECs and mTECs were flow cytometrically isolated by a cell sorter from neonatal thymus of Stat3-f/f mice and Cre-Stat3-f/f mice. Genomic DNA was extracted, and analyze by PCR.
(PDF)

S2 Fig Histological phenotype of thymus of Foxn1-Cre::stat3f/+ mice was indistinguishable from that of Stat3-f/f mice. Immunohistology of cTECs (K8; red) and mTECs (K14; green) in thymus of Foxn1-Cre::Stat3-fl/+ mice and Stat3-fl/fl mice at 9 weeks of age. Scale bars: 400 μm.
(PDF)

S3 Fig Ratio of mTECs is reduced in Foxn1-CKO thymus. Representative flow cytometric profile showing frequencies of individual TEC populations from 8 weeks old Stat3f/f and Foxn1Cre::Stat3f/f mice. The displayed cells were first gated on EpCAM^+^, CD45^-^ (upper panel) and then according to MHC-II (I-A/I-E) and Ly51 expression to highlight medullary (mTEC) and cortical (cTEC) populations. The experiment was performed at the Weizmann institute using Foxn1-Cre knockin mice.
(PDF)

S4 Fig The regenerative potential of K5-Stat3-CKO cTECs is not affected. (A) Experimental design for data presented in panels (B) and (C). Fetal thymi (15 dpc) of K5-Cre::Stat3-fl/fl mice were treated with deoxyguanosine and subsequently transplanted under kidney capsule of wild type mice. After 4 weeks, mice were sacrificed and thymic grafts were examined. (B) Macroscopy of the thymic grafts 4 weeks after bone marrow transplantation. (C) Cryostat sections of thymic grafts were stained with anti-K8 (red) and anti-K14 antibody (green). Sections were counterstained with DAPI (blue). Scale bars: 400 mm.
(PDF)

S5 Fig Normal T cell production in aged Foxn1-Stat3-CKO mice. (A) Immunohistology of cTECs (K8; red) and mTECs (K14; green) in control mice and Foxn1-Cre::Stat3-fl/fl mice at 26 months of age. Scale bars: 400 mm. (B) Quantitative analysis for proportion of mTECs in thymus of control (containing cre-f/+ and f/f, n = 3) and mutant (cre-f/f, n = 6) mice. (C) Flowcytometric profiles of developing thymocytes derived from 22 month old mice. (D) TREC analysis of peripheral T cells from 22 month old mice. (E) Flowcytometric profiles of splenic CD3^+^ cells from 22 month old mice. (F) Flowcytometric profiles of regulatory T cells in thymocytes and in lymphatic CD4^+^ cells from 22 month old mice. (G) Proportion of regulatory T cells in thymocytes and in lymphatic CD4^+^ cells from control (containing cre-f/+ and f/f, n = 3) and mutant (cre-f/f, n = 4) mice at 22 months of age. ns denotes a non-significant difference (P>0.1) in Student’s t test.
(PDF)

S6 Fig HGF-R is not involved in development/maintenance of TECs. (A) Expression of EGF-R and HGF-R in flow cytometrically sorted cTECs and mTECs from wild type mice at one week of age was assessed by RNA seq analysis. (B) Cryostat sections of thymus from control (HGF-R+/+::EGF-Rf/f), HGF-R-CKO (Foxn1-Cre::HGF-Rf/f::EGF-Rf/+), EGF-R-CKO (Foxn1-Cre::HGF-R+/+::EGF-Rf/f), and EGF-R HGF-R-DKO (Foxn1-Cre::HGF-Rf/f::EGF-Rf/f) mice. Scale bars: 400 μm.
(PDF)
