# Supplementary material for: Requirement of Stat3 Signaling in the Postnatal Development of Thymic Medullary Epithelial Cells
Source: PLoS Genet. 2016 Jan 20;12(1):e1005776. doi: 10.1371/journal.pgen.1005776 (PMC4720355; doi:10.1371/journal.pgen.1005776)
Supplement: S1 Fig — cTECs and mTECs were flow cytometrically isolated by a cell sorter from neonatal thymus of Stat3-f/f mice and Cre-Stat3-f/f mice. Genomic DNA was extracted, and analyze by PCR. (PDF) [file pgen.1005776.s001.pdf]

Fig S1

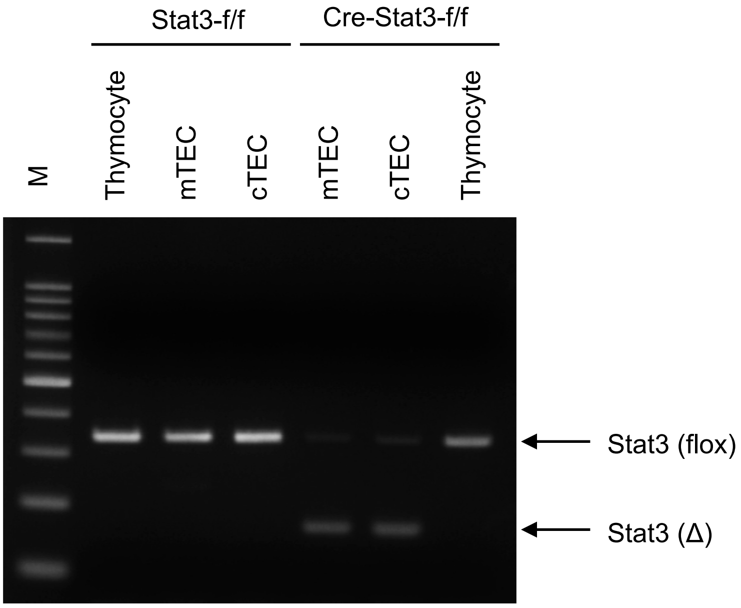

S1 Fig. Stat3 is efficiently deleted in both mTECs and cTECs
cTECs and mTECs were flow cytometrically isolated by a cell sorter from neonatal thymus of Stat3-f/f mice and Cre-Stat3-f/f mice. Genomic DNA was extracted, and analyzed by PCR.
