# Supplementary material for: Requirement of Stat3 Signaling in the Postnatal Development of Thymic Medullary Epithelial Cells
Source: PLoS Genet. 2016 Jan 20;12(1):e1005776. doi: 10.1371/journal.pgen.1005776 (PMC4720355; doi:10.1371/journal.pgen.1005776)
Supplement: S2 Fig — Immunohistology of cTECs (K8; red) and mTECs (K14; green) in thymus of Foxn1-Cre::Stat3-fl/+ mice and Stat3-fl/fl mice at 9 weeks of age. Scale bars: 400 μm. (PDF) [file pgen.1005776.s002.pdf]

Fig S2

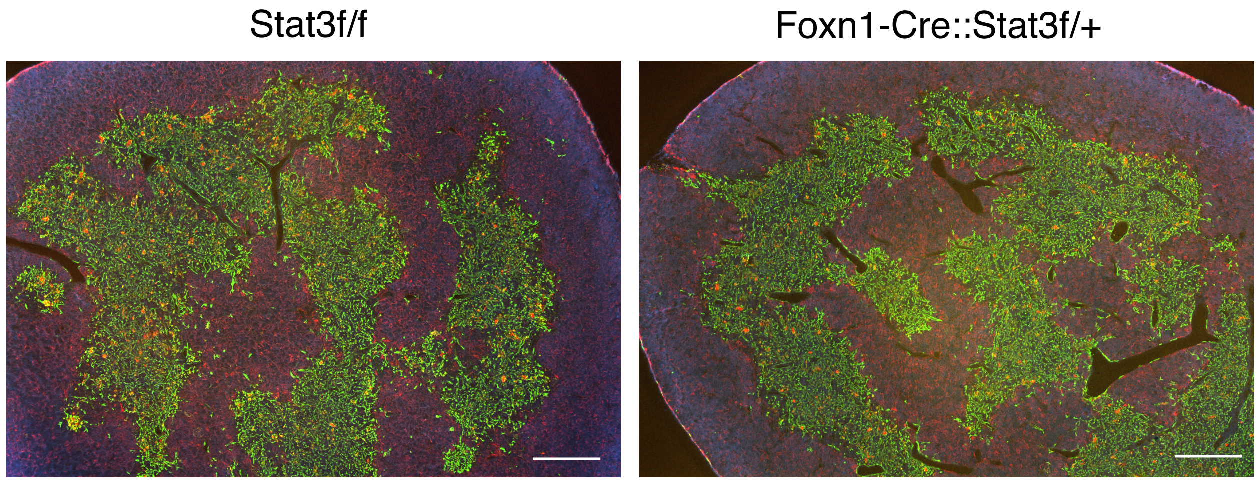

S2 Fig. Histological phenotype of thymus of Foxn1-Cre::stat3f/+ mice was indistinguishable from that of Stat3-f/f mice

Immunohistology of cTECs (K8; red) and mTECs (K14; green) in thymus of Foxn1-Cre::Stat3-fl/+ mice and Stat3-fl/fl mice at 9 weeks of age. Scale bars: 400 µm.
