# Supplementary material for: Requirement of Stat3 Signaling in the Postnatal Development of Thymic Medullary Epithelial Cells
Source: PLoS Genet. 2016 Jan 20;12(1):e1005776. doi: 10.1371/journal.pgen.1005776 (PMC4720355; doi:10.1371/journal.pgen.1005776)
Supplement: S3 Fig — Representative flow cytometric profile showing frequencies of individual TEC populations from 8 weeks old Stat3f/f and Foxn1Cre::Stat3f/f mice. The displayed cells were first gated on EpCAM+, CD45- (upper panel) and then according to MHC-II (I-A/I-E) and Ly51 expression to highlight medullary (mTEC) and cortical (cTEC) populations. The experiment was performed at the Weizmann institute using Foxn1-Cre knockin mice. (PDF) [file pgen.1005776.s003.pdf]

Fig S3

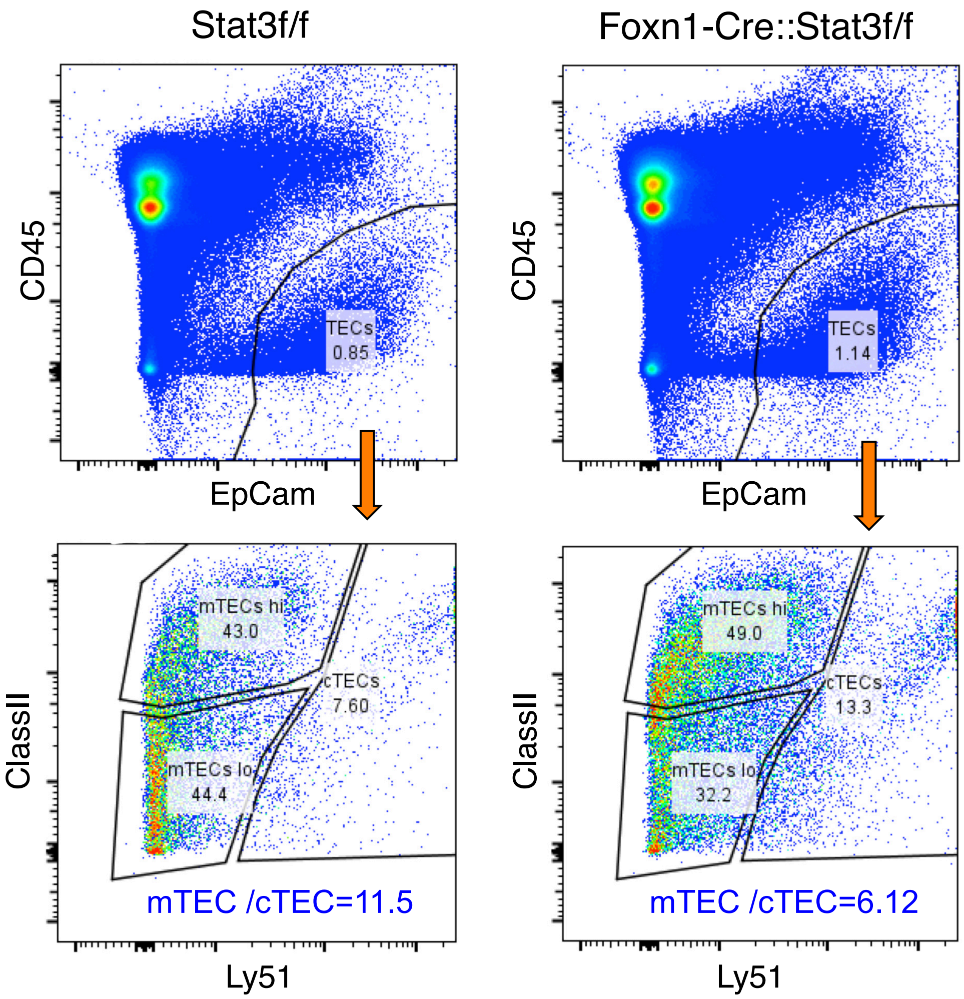

S3 Fig. Ratio of mTECs is reduced in Foxn1-CKO thymus

Representative flow cytometric profile showing frequencies of individual TEC populations from 8 weeks old Stat3f/f and Foxn1Cre::Stat3f/f mice. The displayed cells were first gated on EpCAM$^+$, CD45$^-$ (upper panel) and then according to MHC-II (I-A/ I-E) and Ly51 expression to highlight medullary (mTEC) and cortical (cTEC) populations. The experiment was performed at the Weizmann institute using Foxn1-Cre knockin mice.
