# Supplementary material for: Requirement of Stat3 Signaling in the Postnatal Development of Thymic Medullary Epithelial Cells
Source: PLoS Genet. 2016 Jan 20;12(1):e1005776. doi: 10.1371/journal.pgen.1005776 (PMC4720355; doi:10.1371/journal.pgen.1005776)
Supplement: S4 Fig — (A) Experimental design for data presented in panels (B) and (C). Fetal thymi (15 dpc) of K5-Cre::Stat3-fl/fl mice were treated with deoxyguanosine and subsequently transplanted under kidney capsule of wild type mice. After 4 weeks, mice were sacrificed and thymic grafts were examined. (B) Macroscopy of the thymic grafts 4 weeks after bone marrow transplantation. (C) Cryostat sections of thymic grafts were stained with anti-K8 (red) and anti-K14 antibody (green). Sections were counterstained with DAPI (blue). Scale bars: 400 mm. (PDF) [file pgen.1005776.s004.pdf]

Fig S4

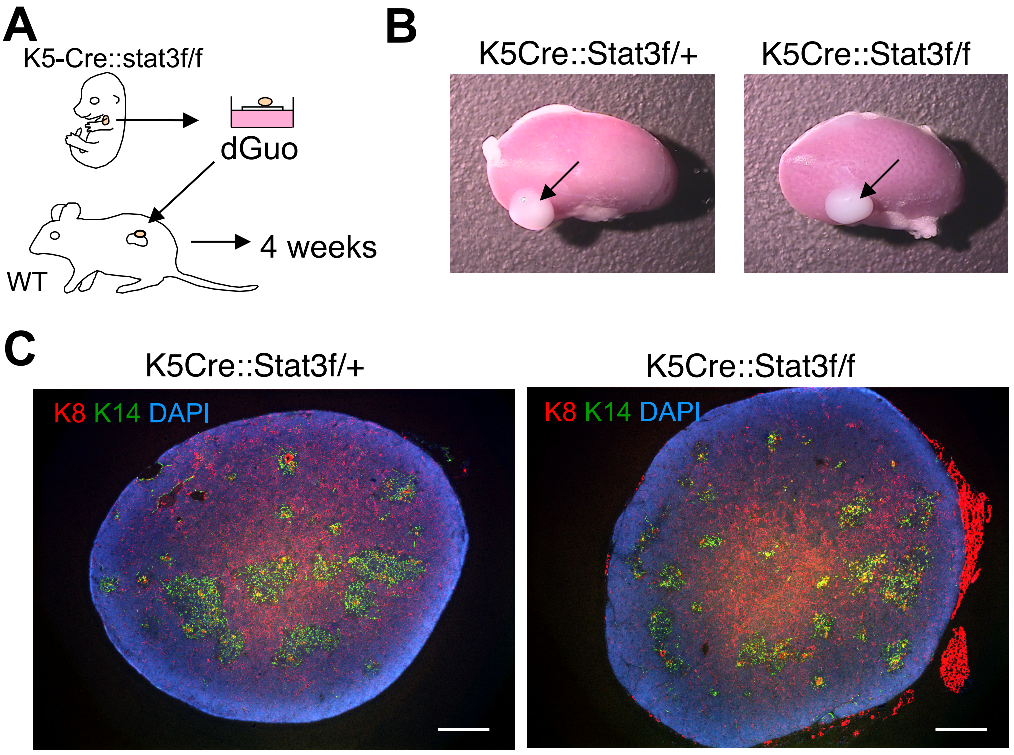

S4 Fig. The regenerative potential of K5-Stat3-CKO cTECs is not affected

(A) Experimental design for data presented in panels (B) and (C). Fetal thymi (15 dpc) of K5-Cre::Stat3-fl/fl mice were treated with deoxyguanosine and subsequently transplanted under kidney capsule of wild type mice. After 4 weeks, mice were sacrificed and thymic grafts were examined.

(B) Macroscopy of the thymic grafts 4 weeks after bone marrow transplantation.

(C) Cryostat sections of thymic grafts were stained with anti-K8 (red) and anti-K14 antibody (green). Sections were counterstained with DAPI (blue). Scale bars: 400 μm.
