# Supplementary material for: Requirement of Stat3 Signaling in the Postnatal Development of Thymic Medullary Epithelial Cells
Source: PLoS Genet. 2016 Jan 20;12(1):e1005776. doi: 10.1371/journal.pgen.1005776 (PMC4720355; doi:10.1371/journal.pgen.1005776)
Supplement: S5 Fig — (A) Immunohistology of cTECs (K8; red) and mTECs (K14; green) in control mice and Foxn1-Cre::Stat3-fl/fl mice at 26 months of age. Scale bars: 400 mm. (B) Quantitative analysis for proportion of mTECs in thymus of control (containing cre-f/+ and f/f, n = 3) and mutant (cre-f/f, n = 6) mice. (C) Flowcytometric profiles of developing thymocytes derived from 22 month old mice. (D) TREC analysis of peripheral T cells from 22 month old mice. (E) Flowcytometric profiles of splenic CD3+ cells from 22 month old mice. (F) Flowcytometric profiles of regulatory T cells in thymocytes and in lymphatic CD4+ cells from 22 month old mice. (G) Proportion of regulatory T cells in thymocytes and in lymphatic CD4+ cells from control (containing cre-f/+ and f/f, n = 3) and mutant (cre-f/f, n = 4) mice at 22 months of age. ns denotes a non-significant difference (P>0.1) in Student’s t test. (PDF) [file pgen.1005776.s005.pdf]

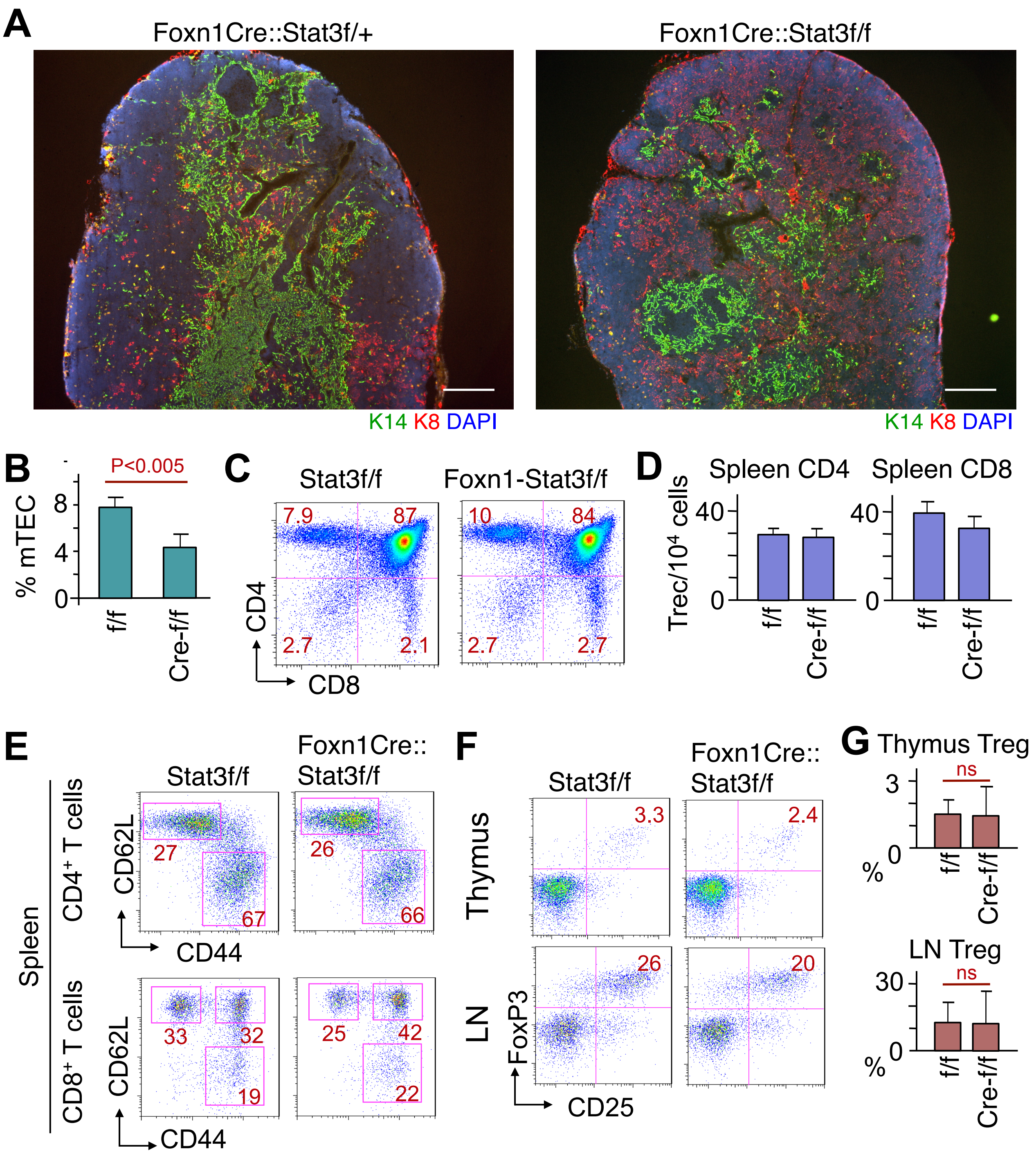

S5 Fig. Normal T cell production in aged Foxn1-Stat3-CKO mice.

(A) Immunohistology of cTECs (K8; red) and mTECs (K14; green) in control mice and Foxn1-Cre::Stat3-fl/fl mice at 26 months of age. Scale bars: 400 µm.

(B) Quantitative analysis for proportion of mTECs in thymus of control (containing cre-f/+ and f/f, n=3) and mutant (cre-f/f, n=6) mice.

(C) Flowcytometric profiles of developing thymocytes derived from 22 month old mice.

(D) TREC analysis of peripheral T cells from 22 month old mice.

(E) Flowcytometric profiles of splenic $CD3^+$ cells from 22 month old mice.

(F) Flowcytometric profiles of regulatory T cells in thymocytes and in lymphatic $CD4^+$ cells from 22 month old mice.

(G) Proportion of regulatory T cells in thymocytes and in lymphatic $CD4^+$ cells from control (containing cre-f/+ and f/f, n=3) and mutant (cre-f/f, n=4) mice at 22 months of age. ns denotes a non-significant difference (P>0.1) in Student's t test.
