# Supplementary material for: Requirement of Stat3 Signaling in the Postnatal Development of Thymic Medullary Epithelial Cells
Source: PLoS Genet. 2016 Jan 20;12(1):e1005776. doi: 10.1371/journal.pgen.1005776 (PMC4720355; doi:10.1371/journal.pgen.1005776)
Supplement: S6 Fig — (A) Expression of EGF-R and HGF-R in flow cytometrically sorted cTECs and mTECs from wild type mice at one week of age was assessed by RNA seq analysis. (B) Cryostat sections of thymus from control (HGF-R+/+::EGF-Rf/f), HGF-R-CKO (Foxn1-Cre::HGF-Rf/f::EGF-Rf/+), EGF-R-CKO (Foxn1-Cre::HGF-R+/+::EGF-Rf/f), and EGF-R HGF-R-DKO (Foxn1-Cre::HGF-Rf/f::EGF-Rf/f) mice. Scale bars: 400 μm. (PDF) [file pgen.1005776.s006.pdf]

Fig S6

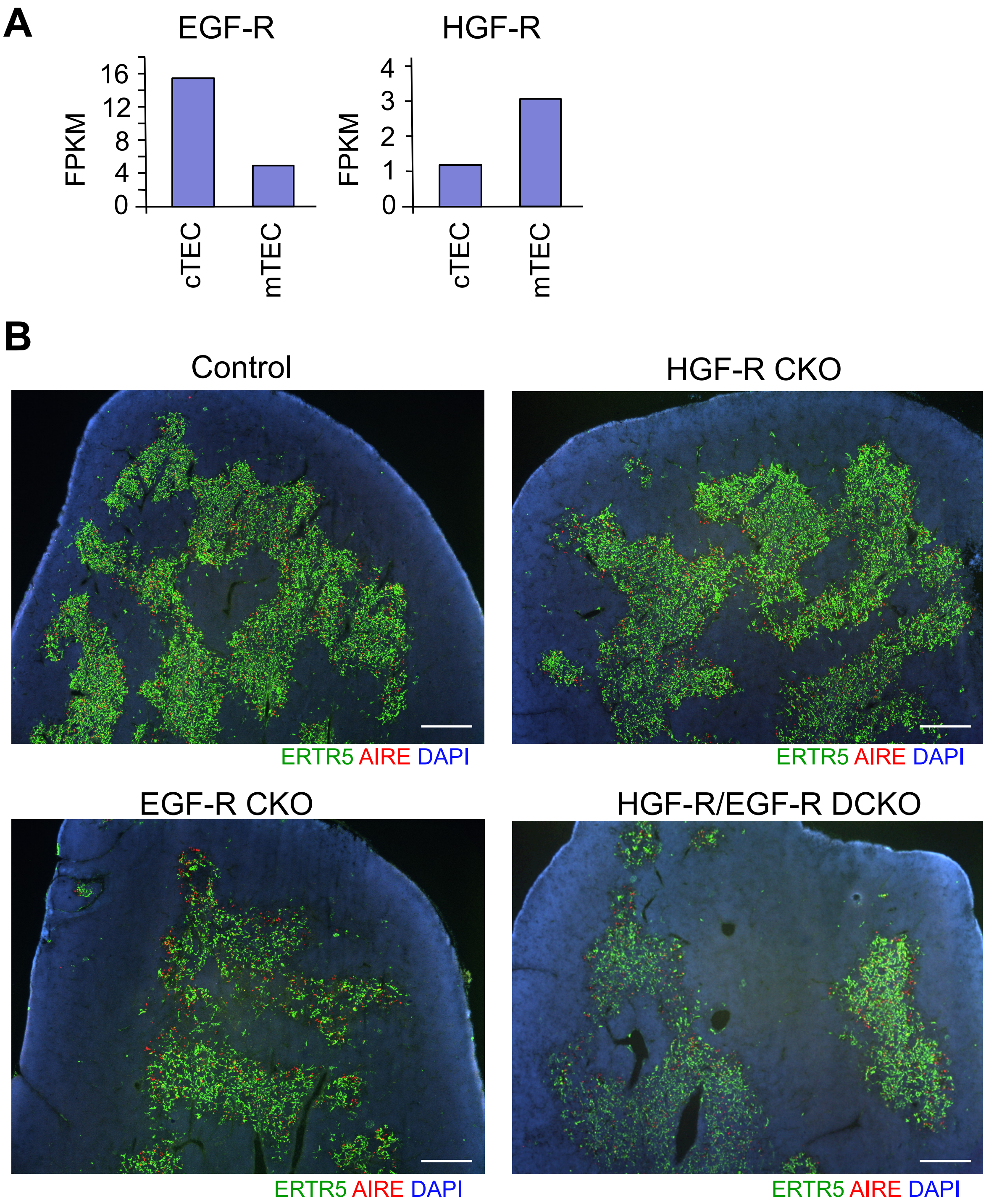

S6 Fig. HGF-R is not involved in development/maintenance of TECs

(A) Expression of EGF-R and HGF-R in flow cytometrically sorted cTECs and mTECs from wild type mice at one week of age was assessed by RNA seq analysis.

(B) Cryostat sections of thymus from control (HGF-R+/+::EGF-Rf/f), HGF-R-CKO (Foxn1-Cre::HGF-Rf/f::EGF-Rf/+), EGF-R-CKO (Foxn1-Cre::HGF-R+/+::EGF-Rf/f), and EGF-R HGF-R-DKO (Foxn1-Cre::HGF-Rf/f::EGF-Rf/f) mice. Scale bars: 400 μm.
